# Modeling the Effects of Elevated Temperature and Weed Interference on Rice Grain Yield

**DOI:** 10.3389/fpls.2021.663779

**Published:** 2021-07-16

**Authors:** Jong-Seok Song, Ji-Hoon Im, Yeon-Ho Park, Soo-Hyun Lim, Min-Jung Yook, Byun-Woo Lee, Jin-Won Kim, Do-Soon Kim

**Affiliations:** ^1^Department of Agriculture, Forestry, and Bioresources, Research Institute of Agriculture and Life Sciences, College of Agriculture and Life Sciences, Seoul National University, Seoul, South Korea; ^2^Institute of Plasma Technology, Korea Institute of Fusion Energy, Gunsan, South Korea; ^3^Mushroom Research Division, Department of Herbal Crop Research, National Institute of Horticultural and Herbal Science, Rural Development Administration, Eumseong, South Korea; ^4^Crop Protection Division, Department of Agro-Food Safety and Crop Protection, National Institute of Agricultural Sciences, Rural Development Administration, Wanju, South Korea

**Keywords:** crop-weed competition, elevated temperature, modeling, late watergrass, water chestnut, rice

## Abstract

A 3-year phytotron study was conducted in Suwon (37.27°N, 126.99°E), Korea, to evaluate and model the effects of elevated temperature on rice-weed competition. The dry weight and the number of panicles in rice were the most susceptible components to weed interference during the early growth of rice, regardless of weed species, while other yield components, including the number of grains, % ripened grain, and 1000-grain weight, were more susceptible to elevated temperature. A rectangular hyperbolic model well demonstrated that rice grain yield was affected by weed interference under elevated temperature, showing that the competitiveness of late watergrass (*Echinochloa oryzicola*) and water chestnut (*Eleocharis kuroguwai*) increased under elevated temperature conditions. Quadratic and linear models well described the effects of elevated temperature on the weed-free rice grain yield and weed competitiveness values of the rectangular hyperbolic model for the two weed species, respectively. Thus, a combined rectangular hyperbolic model incorporated with the quadratic and linear models well demonstrated the effects of elevated temperature and weed interference on rice grain yield across years. Using the combined model and estimated parameters, the rice grain yields were estimated to be 58.9, 48.5, 41.3, and 35.9% of the yields under weed-free conditions for 80 plants m^−2^ of late watergrass and 86.8, 64.3, 51.1, and 42.3% of the yields under weed-free conditions for 80 plants m^−2^ of water chestnut at 1,300, 1,500, 1,700, and 1,900°C·days of accumulated growing degree days (GDD; from transplanting to flowering, 89 days), respectively. The combined model developed in this study can provide an empirical description of both the elevated temperature and weed interference effects on rice yield and can be used for predicting rice grain yields due to weed interference under future elevated temperature conditions.

## Introduction

According to the Representative Concentration Pathways (RCP) scenarios presented by the fifth assessment report (AR5) of the Intergovernmental Panel on Climate Change (IPCC), anthropogenic climate change challenges current and future global crop production due to the global warming exceeding 1.4°C (RCP6.0) and 4.8°C (RCP8.5) of mean air temperature in the 1980s by the 2080s, along with unprecedented extreme heat stress and high potential CO_2_ fertilization effects (Intergovernmental Panel on Climate Change [(IPCC)], 2013; Deryng et al., 2014). Rice is mainly cultivated in areas with tropical and temperate climates located between 53° northern latitude (Heilongjiang, China) and 35° southern latitude (New South Wales, Australia) (Koo et al., 2013; Kraehmer et al., 2016). The projected warming by the 2080s will have more serious consequences for rice production. For example, exposure to extreme events will reduce rice yield by damaging the development of grain spikelets during the anthesis and grain filling stages of rice (Welch et al., 2010; Nguyen et al., 2014; Sánchez et al., 2014; Jung et al., 2015; Lee et al., 2015). Even worse, the projected warming will negatively affect rice yield with increasing weed competitiveness due to more enhanced weed biomass compared with the biomass of rice (Alberto et al., 1996).

Late watergrass (*Echinochloa oryzicola* Vasing.) and water chestnut (*Eleocharis kuroguwai Ohwi*) are troublesome weed species that inhabit rice paddy fields, mainly in East Asian countries, including Korea, Japan, and Vietnam (Kraehmer et al., 2016; Song et al., 2016, 2017a). These weed species cause significant yield loss of rice due to the higher canopy height of late watergrass and stronger root competitiveness of water chestnut than those of rice during the entire growing season (Yamasue, 2001; Moon et al., 2010; Song et al., 2016). Even when herbicides are applied, late watergrass and water chestnut cannot always be controlled due to herbicide-resistant late watergrass (Fischer et al., 2000; Song et al., 2017a) and the tuberization characteristics of water chestnut in deep soil (Watanabe, 2011; Moon et al., 2014; Song et al., 2016). Late watergrass is a representative C4 weed species that can adapt to some environmental changes in rice cultivation, showing various adaptive characteristics, such as an enhanced photosynthetic capacity under high temperature (Bouhache and Bayer, 1993), enhanced tolerance to salinity (Nguyen et al., 2005), and germination and seedling growth under anaerobic conditions (Kennedy et al., 1980; Nah et al., 2015). Water chestnut can also adapt to such environmental conditions, although it is a C3 weed species (Ueno and Takeda, 1992) that shows tuber propagation under submerged soil conditions (Kobayashi and Ueki, 1983; Chun and Shin, 1994) and fast growth and high biomass under elevated temperatures (Kim et al., 2010). If these weed species are not properly managed under future climate change, then they will become more competitive against rice, resulting in greater yield losses than under current climate conditions.

Many studies have focused on the potential effects of climate change on rice production (Bachelet and Gay, 1993; Kim et al., 1996; Matsui et al., 1997; Matthews et al., 1997; Sheehy et al., 2006a; Mahajan et al., 2012; Nguyen et al., 2014) and paddy weed adaptation (Kim et al., 2010; Park et al., 2010; Rodenburg et al., 2011; Bir et al., 2014; Ramesh et al., 2017), but, relatively, few studies have examined the impacts of climate change on weed-rice competition. Previous studies reported the effects of elevated CO_2_ concentrations on the competitive interactions between weeds and rice (Alberto et al., 1996; Ziska et al., 2010; Zeng et al., 2011) and the effects of elevated CO_2_ or air temperature on the interactions between weeds and other crops, including soybeans (Flint and Patterson, 1983; Patterson et al., 1984; Ziska, 2000, 2001; Davis and Ainsworth, 2012), wheat (Thompson and Woodward, 1994), cotton (Flint et al., 1983), and sorghum (Ziska, 2003). A modeling study on the impacts of climate change and weed interference on crop yield under field conditions has not been reported to our knowledge (Ramesh et al., 2017). Late watergrass and water chestnut are dominant in rice cultivation in Korea and Japan but have not been investigated for their competitive effects on rice yield under future climate change. The evaluation of both the effects of climate change and weed interference on rice is essential to support decision-making for weed management and to establish effective weed management practices under the changing climate conditions. Although climate change can be induced by both elevated temperature and elevated CO_2_, it is difficult to evaluate and model both effects on rice-weed competition. Considering that plant growth is more sensitive to temperature than elevated CO_2_, it will be more effective for establishing weed management practices in rice under future climate conditions to evaluate and model the effects of elevated temperature on rice-weed competition.

Therefore, this study was conducted to evaluate the effects of elevated temperature on late watergrass-rice and water chestnut-rice competition and to develop a model to predict rice yield in the presence of weed interference under elevated temperature conditions.

## Materials and Methods

### Pot Experiments Under Phytotron

Pot experiments under phytotrons were conducted in 2014, 2015, and 2016 at the experimental farm station of Seoul National University, Suwon (37.27°N, 126.99°E), Korea, to evaluate the temperature effects on late watergrass-rice and water chestnut-rice competition. As shown in Table 1, four sunlit temperature-controlled phytotrons were adjusted to the target temperature conditions, ambient (A), A+1.5, A+3.0, and A+5.0°C, by using a heating and ventilation control system linked to a CR10x data logger (Camp-bell Sci., USA) (Nguyen et al., 2014; Jung et al., 2015; Kim et al., 2017; Kim and Lee, 2019). The ambient temperature was maintained with no sidewalls and only plastic film-covered roofs in a plastic house. The ambient temperature differed in different years (Supplementary Figure 1); thus, it was necessary to convert the daily mean air temperature (*T*) into accumulated growing degree days (*GDD*) based on the base temperature (*T*_*base*_) (McMaster and Wilhelm, 1997) as follows:

(1) $$\begin{array}{l} GDD=\left(T-T_{base}\right)\times days \end{array}$$

The base temperature (*T*_*base*_) was taken as 10°C, which is commonly used in Korean rice-growing systems (e.g., Lee et al., 2015). Twenty-day-old rice seedlings (*Oryza sativa* cv. Chucheong) were transplanted into 30-cm-deep plastic pots (0.1 m^2^), containing paddy soil at a density of 30 hills m^−2^, which is equivalent to ~90 rice seedlings m^−2^, on June 1 over 3 years. The paddy soil in the pot had a loam texture with an organic matter content of 19.5 g kg^−1^, total nitrogen concentration of 1.10 g kg^−1^, ${\text{NH}}_{4}^{+}$-N concentration of 2.29 mg kg^−1^, and ${\text{NO}}_{3}^{-}$-N concentration of 288.97 mg kg^−1^. Late water grass and water chestnut were manually sown on the paddy soil immediately after harrowing, randomly thinned to the target plant density for individual weed species immediately after rice transplanting and grown around rice plants until harvest at each of the four sunlit temperature-controlled phytotrons. The target densities for competition between rice and weed species were 0, 10, 20, 40, and 80 plants m^−2^ in 2014 and 0, 20, 40, 80, and 160 plants m^−2^ in 2015 and 2016 for both weed species. Other weed species were removed manually. Fertilization was performed with the basal dressing of N–P_2_O_5_-K_2_O = 55–45–45 kg ha^−1^ before harrowing and two sequential top-dressings of 27.5 kg ha^−1^ of N at both rice tillering and panicle initiation stages. Other cultivation practices, such as irrigation and pest management, were performed, following the standard practice of the Rural Development Administration of Korea (RDA) (Rural Development Administration [RDA], 2014). All the pots were arranged in a completely randomized block design with three replicates in each phytotron.

**Table 1:** Mean air temperature during the period from transplanting (June 1) to tillering (August 10), during the period from transplanting (June 1) to flowering (August 28), and during the whole growth period (June 1 to October 15) at four sunlit temperature-controlled phytotrons adjusted to the target temperature conditions, such as ambient (A), A+1.5°C, A+3.0°C, and A+5.0°C, in 2014, 2015, and 2016.

**Year**	**Growth stage**	**Mean air temperature (°C)**				**Growing degree days (°C·days)**			
		**A**	**A+1.5**	**A+3.0**	**A+5.0**	**A**	**A+1.5**	**A+3.0**	**A+5.0**
2014	Transplanting to tillering	24.6	26.1	27.2	28.7	1,034	1,146	1,224	1,329
	Transplanting to flowering	24.1	25.5	26.7	28.3	1,288	1,426	1,525	1,658
	Transplanting to maturity	23.1	24.6	25.8	27.2	1,791	2,002	2,158	2,352
2015	Transplanting to tillering	25.7	27.1	28.6	30.4	1,113	1,214	1,318	1,448
	Transplanting to flowering	26.4	27.8	28.9	30.9	1,408	1,534	1,659	1,825
	Transplanting to maturity	24.1	25.5	26.9	28.7	1,930	2,129	2,313	2,562
2016	Transplanting to tillering	25.7	27.4	28.8	30.6	1,117	1,233	1,332	1,460
	Transplanting to flowering	29.0	30.8	32.1	33.7	1,460	1,608	1,729	1,886
	Transplanting to maturity	23.8	25.2	26.6	28.2	1,890	2,081	2,276	2,490

In October of each year, rice was harvested at maturity to assess the yield components, including dry weight, number of panicles, number of grains, percentage of ripened grains, and thousand-grain weight. The grain weight and moisture content were also assessed, and the grain yield was calculated by adjusting the grain moisture content to 12%.

### Model Development

A rectangular hyperbolic model (equation 2; Cousens, 1985; Kim et al., 2002; Song et al., 2017b) was used to describe the relationship between rice grain yield (*Y*) and initial single weed density (*X*) under different air temperature conditions.

(2) $$\begin{array}{l} Y=\frac{Y_{o}}{1+\beta X} \end{array}$$

where *Y*_*o*_ is the weed-free rice grain yield (t ha^−1^), and β is a measure of weed competitiveness. When rice and a single weed were grown in a mixture under elevated temperature, the parameters *Y*_*o*_ and β for the model can change with the level of elevated temperature. Their relationships under elevated temperature are slightly unknown and thus must be parameterized separately at each level of elevated temperature (*i*) as follows:

(3) $$\begin{array}{l} Y=\frac{Y_{oi}}{1+\beta_{i}X} \end{array}$$

The parameter *Y*_*oi*_ was further investigated based on the increasing air temperature level (*i*). A quadratic model was used to describe the relationship between the elevated temperature and the resultant rice grain yield (e.g., Sheehy et al., 2006a). Thus, equation 3 can be rewritten into equation 4 with the quadratic model.

(4) $$\begin{array}{l} Y=\frac{a+bGDD+cGDD^{2}}{1+\beta_{i}X} \end{array}$$

where *a, b*, and *c* are unknown parameters for weed-free rice grain yield, and *GDD* is the accumulated growing degree days under elevated temperature. The parameter β_*i*_ may also change with an increasing air temperature level (*i*). A linear model was used to describe the relationship between elevated temperature and resultant weed competitiveness (e.g., Kim et al., 2006). Equation 4 was further modified into equation 5 with the linear model.

(5) $$\begin{array}{l} Y=\frac{a+bGDD+cGDD^{2}}{1+\left(l+mGDD\right)X} \end{array}$$

where *l* and *m* are unknown parameters for weed competitiveness. Equations 4 and 5 were tested to determine whether the quadratic and linear models were appropriate for the change in weed-free grain yield and weed competitiveness, respectively, with GDD resulting from elevated temperature.

### Statistical Analyses

All of the data were initially subjected to three-way analysis of variance (ANOVA). For weed biomass, rice yield components, and grain yield, data were analyzed with year, elevated temperature, and weed density as main factors and the replicate as a random factor. Correlation analyses were conducted for their relationships among yield components, elevated temperature, and weed density to evaluate the effects of elevated temperature and weed interference on the rice yield components and grain yield. Nonlinear regression analyses were also conducted to fit the rectangular hyperbolic model (equation 3) to the yield data corresponding to the plant density of each weed species for each air temperature condition. The quadratic and linear models were regressed on weed-free rice grain yield (*Y*_*o*_) and weed competitiveness (β) affected by elevated temperature in the pooled 3-year data, respectively. Then, by incorporating the models selected for the two parameters, *Y*_*o*_ and β, into the rectangular hyperbolic model (equations 4 and 5), the combined models were evaluated, using the *F*-test (equation 6).

(6) $$\begin{array}{l} F=\frac{\frac{RSS_{i+1}-RSS_{i}}{df_{i+1}-df_{i}}}{\frac{RSS_{f}}{df_{f}}} \end{array}$$

where *RSS* and *df* are the residual sum of the square and the degree of freedom, respectively; *i* + 1 is the model one-step reduced from its predecessor (*i*); and *f* is the full model. If the *F*-value was lower than the tabulated *F*-value (a 5% level) with (*df*_*i*+1_-*df*_*i*_, *df*_*f*_) degrees of freedom, then the reduced model was accepted. The performance of all the models was evaluated by the pseudo-R^2^ of the models and the root mean square error (RMS) of the prediction. All the statistical analyses were conducted, using Genstat (Genstat Committee, 2002).

## Results

### Effects of Elevated Temperature and Weed Interference on Rice

**Figure 1:**
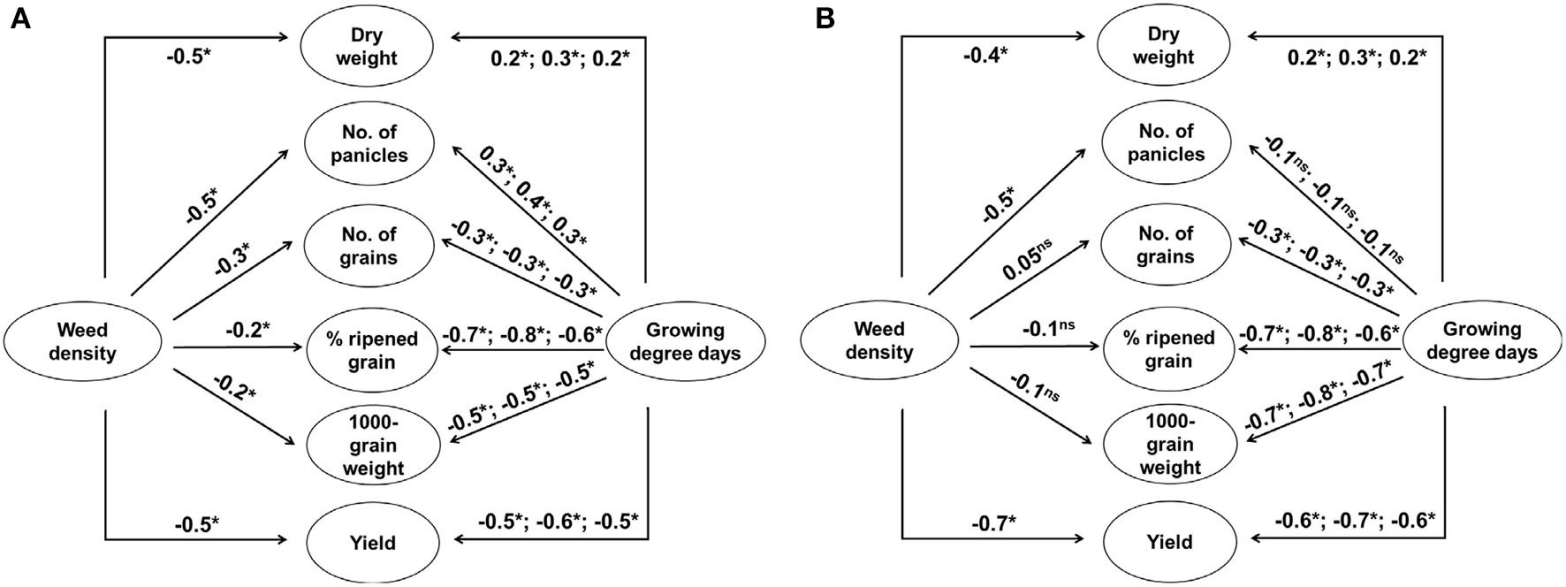
Schematic representations of the correlations among growing degree days (transplanting to tillering, transplanting to flowering, and transplanting to maturity), plant densities of late watergrass **(A)** and water chestnut **(B)**, rice yield components (dry weight, number of panicles, number of grains, % ripened grain, 1,000-grain weight), and rice grain yield over 3 years. Significance is indicated as follows: ^ns^, not significant; *, *P* < 0.05.

The three-way ANOVA revealed that both weed and rice were significantly affected by not only elevated temperature and weed density but also the year of experiment in a variety of ways (Supplementary Table 1). Late watergrass was affected solely by year and its plant density, while water chestnut was affected by each of the three main factors and the interaction of year and its plant density. In the case of rice, year, elevated temperature, and weed density interactively affected rice during the entire growing season. In particular, weed density more strongly affected rice vegetative growth and its yield component (e.g., number of panicles) determined at earlier growth stages, while the interactive effect of year and elevated temperature more strongly affected rice reproductive growth and its yield components (e.g., number of grains, % ripened grain, 1,000-grain weight) determined at later growth stages (Supplementary Table 1). As the ambient temperature differed in different years (Supplementary Figure 1), accumulated growing degree days (GDD) was expressed as a function of year and elevated temperature. Correlation analyses described the relationships between weed interference and rice yield components and between GDD and rice yield components across years (Figure 1). Dry weight and the number of panicles were negatively affected by weed density regardless of weed species, while the other yield components, including the number of grains, % ripened grains, and 1,000-grain weight, were not always affected by weed density (Figure 1). In contrast, the number of grains, % ripened grains, and 1,000-grain weight in the rice yield components were negatively affected by GDD, while the number of panicles was not always affected by GDD. Thus, rice grain yield was associated with weed interference and GDD, especially up to the flowering stage of rice.

**Figure 2:**
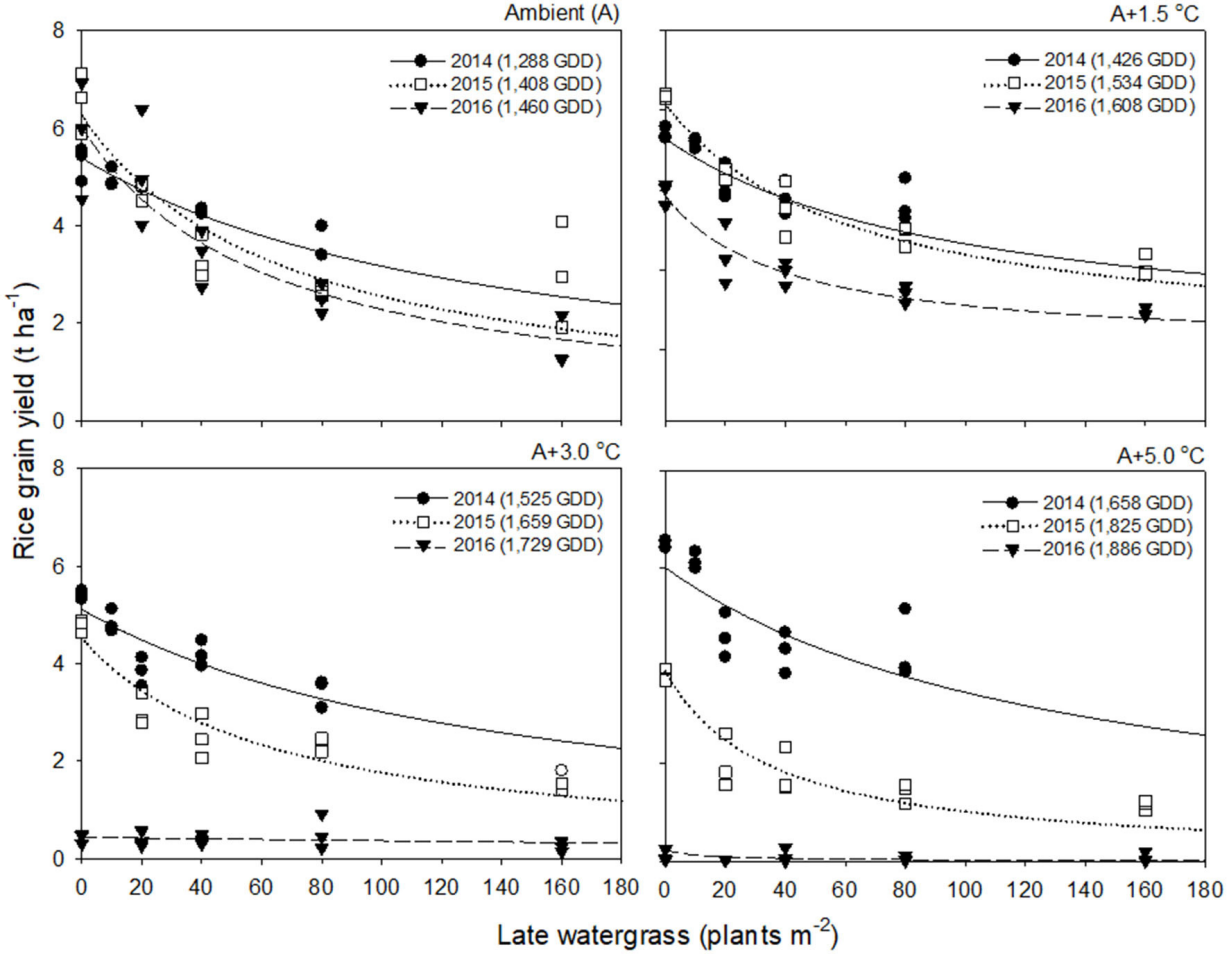
Rice grain yield as a function of the weed density of late watergrass during accumulated growing degree days (GDD; from transplanting to flowering, 89 days) at four sunlit temperature-controlled phytotrons adjusted to ambient (A), A+1.5°C, A+3.0°C, and A+5.0°C in 2014 (•), 2015 (□), and 2016 (▾). The lines are fitted values calculated, using the rectangular hyperbolic model (equation 3) and parameter estimates (Table 2).

**Figure 3:**
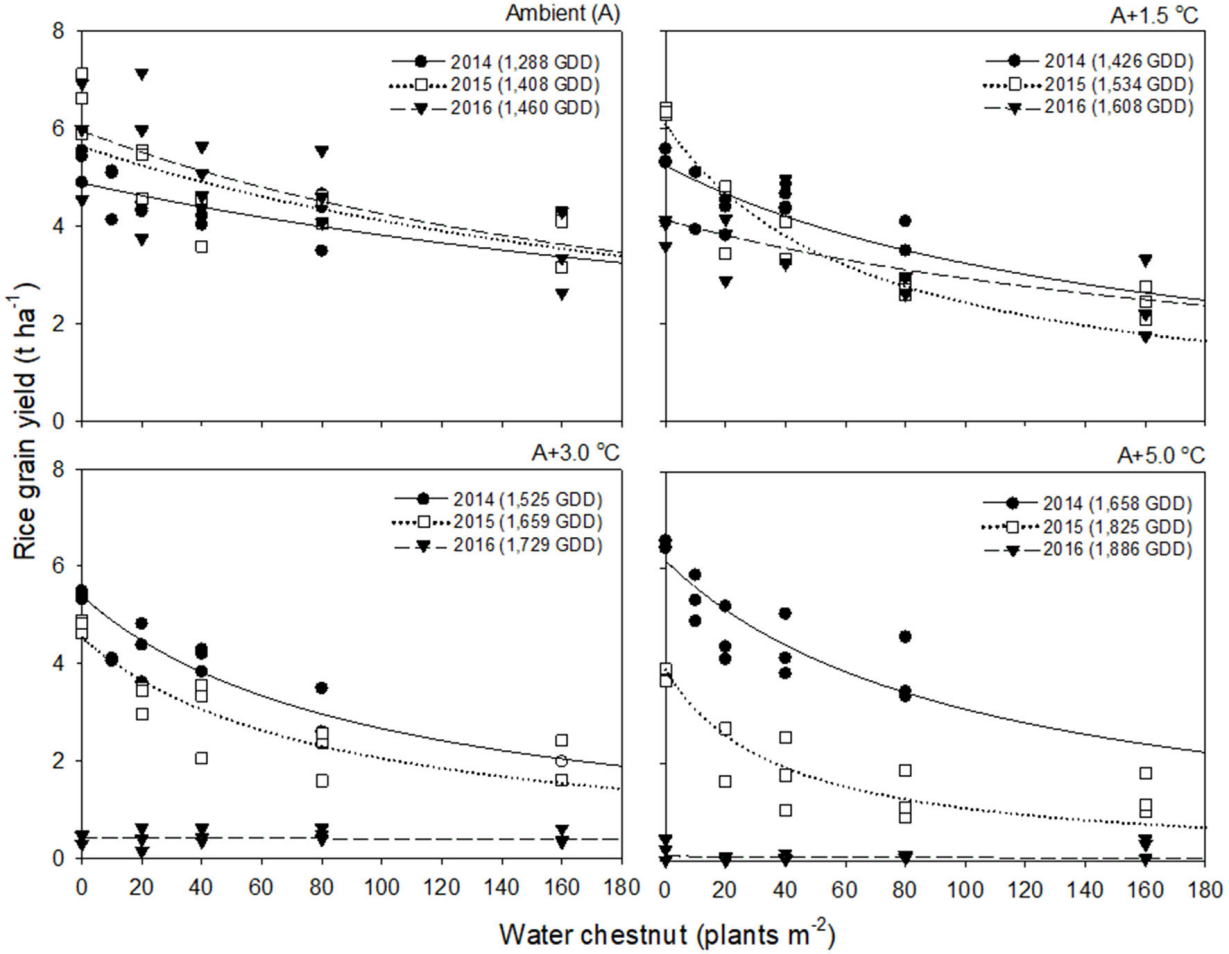
Rice grain yield as a function of the weed density of water chestnut during accumulated growing degree days (GDD; from transplanting to flowering, 89 days) at four sunlit temperature-controlled phytotrons adjusted to ambient (A), A+1.5°C, A+3.0°C, and A+5.0°C in 2014 (•), 2015 (□), and 2016 (▾). The lines are fitted values calculated, using the rectangular hyperbolic model (equation 3) and parameter estimates (Table 2).

Rice grain yield in 2016 was much lower than those in 2014 and 2015 (Figures 2, 3). This finding was attributed to the significant reduction in % ripened grains by high air temperatures over 30°C during the heading and flowering periods (Supplementary Figure 2). The % ripened grain was dependent on the ambient air temperature observed over 3 years, so it decreased with increasing ambient air temperature, giving 97.3, 96.0, and 88.8% ripened grains in 2014, 2015, and 2016, when the ambient (A) air temperature was 24.1, 26.4, and 29.0°C, respectively (Supplementary Figure 2). Elevated temperature further reduced the % ripened grain in combination with the ambient air temperature. In 2014, when the ambient air temperature was low, the % ripened grain remained high even at an elevated temperature of A+5.0°C, giving 93.4% ripened grains. In contrast, in 2016, when the ambient air temperature was high, the % ripened grain decreased significantly, resulting in 50.2, 4.9, and 1.7% ripened grains at elevated temperatures of A+1.5, A+3.0, and A+5.0°C, respectively. Due to the high ambient air temperature of 29.0°C in 2016, an elevated temperature of A+1.5°C resulted in over 30.5°C exposure during the heading and flowering periods (Table 1). This finding thus clearly demonstrates that the % ripened grain depends on the air temperature resulting from the combined ambient + elevated temperature that rice was exposed to during the heading and flowering periods, leading to a significant reduction of the % ripened grain if the combined ambient + elevated temperature is over 30°C.

### Response of Weed-Free Rice Grain Yield and Weed Competitiveness to Elevated Temperature

**Figure 4:**
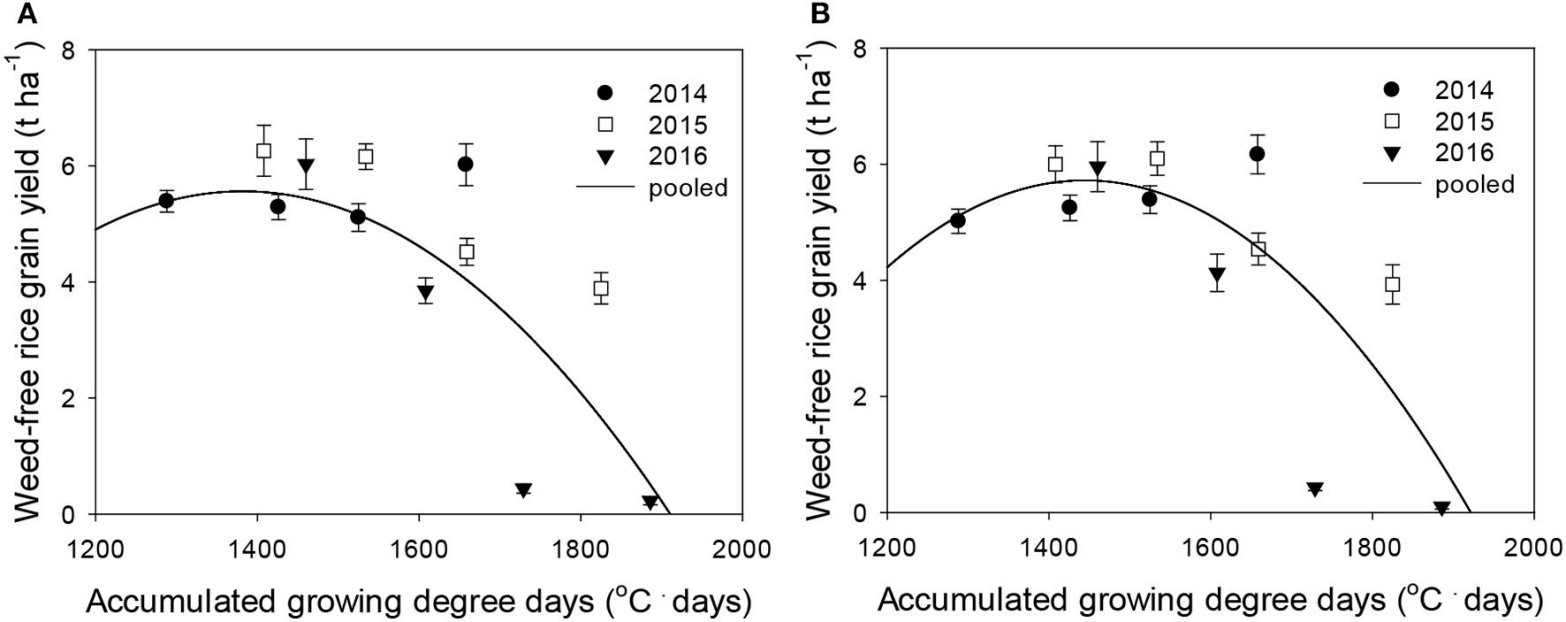
Weed-free rice grain yield (*Y*_*o*_) as a function of the accumulated growing degree days (from transplanting to flowering, 89 days) at four sunlit temperature-controlled phytotrons adjusted to ambient, +1.5°C, +3.0°C, and +5.0°C in 2014, 2015, and 2016. The lines are fitted values calculated, using the quadratic model and parameter estimates (Supplementary Table 2) in the pooled 3-year data of late watergrass **(A)** and water chestnut **(B)**.

**Figure 5:**
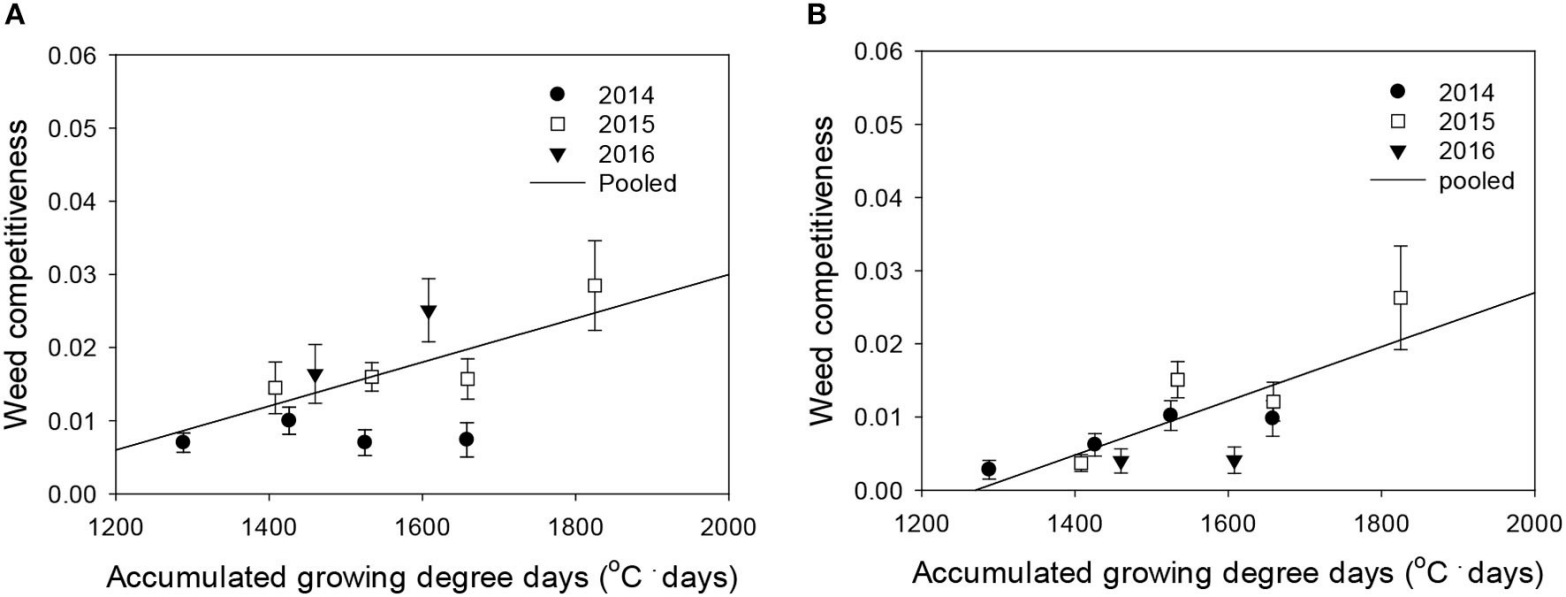
Weed competitiveness (β) as a function of the accumulated growing degree days (from transplanting to flowering, 89 days) at four sunlit temperature-controlled phytotrons adjusted to ambient, +1.5°C, +3.0°C, and +5.0°C in 2014, 2015, and 2016. The lines are fitted values calculated, using the linear model and parameter estimates (Supplementary Table 3) in the pooled 3-year data of late watergrass **(A)** and water chestnut **(B)**.

Rice grain yield was regressed on single-species weed density at different GDDs, using a rectangular hyperbolic model (Table 2). The yield data for each species showed year-to-year variations due to the different GDDs (from transplanting to flowering, 89 days) at four sunlit temperature-controlled phytotrons adjusted to A, A+1.5°C, A+3.0°C, and A+5.0°C in 2014, 2015, and 2016 (Figures 2, 3). Quadratic and linear models were regressed on the weed-free rice grain yield and weed competitiveness affected by GDD (from transplanting to flowering, 89 days) over 3 years (Figures 4, 5). For the weed-free rice grain yield, the pooled value increased by up to 5.56 t and 5.72 t ha^−1^ for late watergrass and water chestnut, respectively, and, thereafter, decreased (Figure 4). Weed-free grain yield was associated with a lower percentage of ripened grains due to the elevated temperature during its heading and flowering periods (Supplementary Figure 2). For weed competitiveness, the pooled value exhibited a linear increase regardless of weed species with increasing GDD (Figure 5). Weed competitiveness was associated with a reduced number of panicles as a result of rice-weed competition during the period from the transplanting to tillering stages of rice. The weed competitiveness for the number of panicles showed a similar increase to the competitiveness for rice grain yield (Supplementary Figure 3).

**Table 2:** Parameter estimates for the rectangular hyperbolic model of rice grain yield as a result of single-weed interference caused by late watergrass and water chestnut at accumulated growing degree days (from transplanting to flowering, 89 days) in 2014, 2015, and 2016.

**Year**	**Growing degree days**	**Parameter estimates for late watergrass^*a*^**				**Parameter estimates for water chestnut^a^**			
		***y***_***o***_	**β**	**RMS**	**Pseudo-R^2^**	***y***_***o***_	**β**	**RMS**	**Pseudo-R^2^**
2014	1288	5.39 (0.186)	0.0070 (0.00132)	0.37	0.81	5.02 (0.209)	0.0034 (0.00123)	0.43	0.47
	1426	5.29 (0.216)	0.0100 (0.00185)	0.41	0.81	5.25 (0.221)	0.0062 (0.00153)	0.44	0.68
	1525	5.11 (0.239)	0.0070 (0.00178)	0.47	0.66	5.39 (0.237)	0.0102 (0.00203)	0.45	0.81
	1658	6.02 (0.360)	0.0074 (0.00234)	0.70	0.53	6.17 (0.334)	0.0098 (0.00243)	0.64	0.69
2015	1408	6.26 (0.438)	0.0145 (0.00353)	0.83	0.72	6.00 (0.318)	0.0046 (0.00130)	0.70	0.61
	1534	6.16 (0.221)	0.0160 (0.00195)	0.41	0.93	6.10 (0.289)	0.0151 (0.00247)	0.54	0.87
	1659	4.52 (0.232)	0.0157 (0.00275)	0.44	0.85	4.54 (0.274)	0.0121 (0.00266)	0.53	0.77
	1825	3.89 (0.270)	0.0285 (0.00613)	0.48	0.81	3.93 (0.338)	0.0263 (0.00709)	0.60	0.75
2016	1460	6.03 (0.435)	0.0164 (0.00401)	0.81	0.80	5.96 (0.432)	0.0040 (0.00165)	0.97	0.42
	1608	3.85 (0.211)	0.0251 (0.00432)	0.38	0.89	4.13 (0.323)	0.0041 (0.00181)	0.73	0.39
	1729	0.44 (0.079)	0.0021 (0.00317)	0.19	0.02	0.43 (0.052)	0.0005 (0.00129)	0.14	0.06
	1886	0.23 (0.071)	0.0842 (0.08918)	0.12	0.15	0.10 (0.040)	0.0039 (0.00121)	0.14	0.08

a *A rectangular hyperbolic model $\left(Y=\frac{Y_{o}}{1+\beta X}\right)$, where Y_0_ is the weed-free rice grain yield (t ha^−1^), β is a measure of weed competitiveness, and X is the initial single weed density under different air temperature conditions. The numbers in parentheses are standard errors (d.f. = 13)*.

### Combined Model for How Rice Grain Yield Is Affected by Elevated Temperature and Weed Interference

The quadratic and linear models were sequentially incorporated into the rectangular hyperbolic model to describe the relationships between weed-free rice grain yield and GDD and weed competitiveness and GDD, respectively. When the relationship between weed-free rice grain yield (*Y*_*oi*_) versus GDD was replaced by the quadratic model, there was a significant difference between equation 3 (the rectangular hyperbolic model) and equation 4 (the intermediate model) to describe the rice grain yield affected by late watergrass and water chestnut under elevated temperature (Table 3). When the relationship between weed competitiveness (β_*i*_) and GDD was replaced by the linear model, there was a significant difference between equation 4 (the intermediate model) and equation 5 (the combined rectangular hyperbolic model) in describing the same rice grain yield (Table 3). Although there was a significant difference between the tested models, the quadratic and linear models seemed to well describe the weed-free rice grain yield and weed competitiveness affected by GDD, respectively, with a small root mean square error and high pseudo-R^2^ values (Supplementary Tables 2, 3). Thus, these models can be incorporated in the rectangular hyperbolic model to describe the effects of elevated temperature on weed-free rice grain yield and weed competitiveness. Our results revealed that the combined rectangular hyperbolic model could describe both the elevated temperature and weed competition effects on rice grain yield regardless of weed species.

**Table 3:** A summary of the nonlinear regression analysis and lack-of-fit test to compare the models for the regression of rice grain yield as a result of single-weed interference caused by late watergrass and water chestnut at accumulated growing degree days (from transplanting to flowering, 89 days) in the pooled 3-year data.

**Weed species**	**Equation^a^**	**Residual**		**Number of parameters**	**Test statistics**	
		**df**	**SS**		**Comparison**	**F-value**
	Rice grain yield					
Late watergrass	[3]	118	37.19	20		
	[4]	125	51.88	13	[4]-[3]	6.66*
	[5]	133	92.91	5	[5]-[4]	16.27*
	Rice grain yield					
Water chestnut	[3]	117	47.57	20		
	[4]	124	62.47	13	[4]-[3]	5.24*
	[5]	132	84.58	5	[5]-[4]	6.80*

a *Equation 3 is a rectangular hyperbolic model ($Y=\frac{Y_{0i}}{1+\beta_{i}X}$); equation 4 is an intermediate model $\left(Y=\frac{a+bGDD+cGDD^{2}}{1+\beta_{i}X}\right)$; equation 5 is a combined rectangular hyperbolic model $\left(Y=\frac{a+bGDD+cGDD^{2}}{1+\left(l+mGDD\right)X}\right)$*.

**Figure 6:**
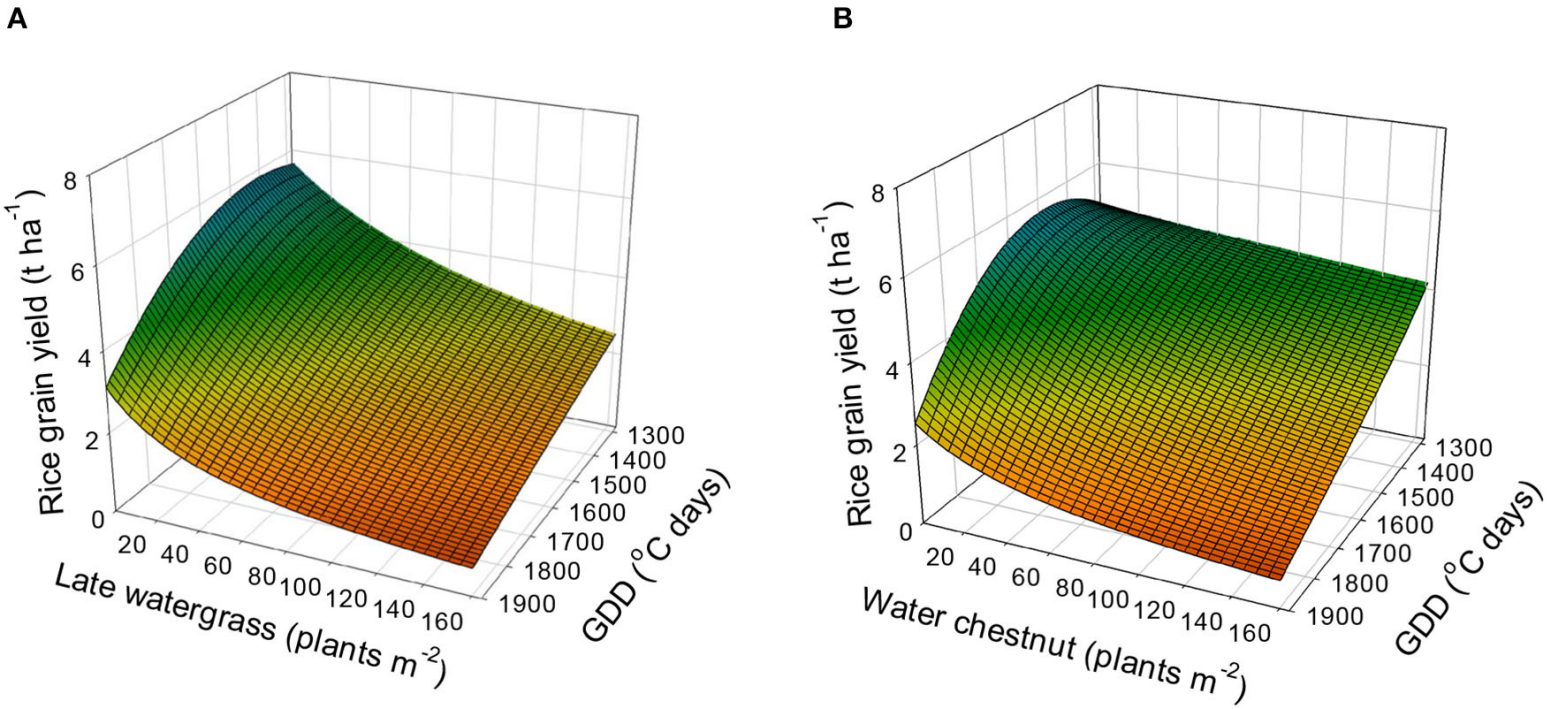
Predicted rice grain yield as a function of the weed density and accumulated growing degree days (GDD; from transplanting to flowering, 89 days) under late watergrass **(A)** and water chestnut **(B)** interference and elevated temperature conditions. The mesh is fitted values calculated, using the combined rectangular hyperbolic model (equation 5) and parameter estimates (Table 4).

Using equation 5 (the combined rectangular hyperbolic model) and the estimated parameters (Table 4), the rice grain yield was predicted (Figure 6). The rice grain yields without interference were estimated to be 5.1 to 5.7 t, 5.6 to 5.8 t, 4.8 to 4.9 t, and 2.5 to 3.1 t ha^−1^ at 1,300, 1,500, 1,700, and 1,900°C days of GDD (from transplanting to flowering, 89 days), respectively. When late watergrass was interfered with 80 plants m^−2^, the model predicted that the rice grain yields would be reduced by 58.9, 48.5, 41.3, and 35.9% of the yields under weed-free conditions at 1,300, 1,500, 1,700, and 1,900°C days of GDD (from transplanting to flowering, 89 days), respectively. When water chestnut was applied at 80 plants m^−2^, the rice grain yields were reduced by 86.8, 64.3, 51.1, and 42.3% of the yields under weed-free conditions at 1,300, 1,500, 1,700, and 1,900°C days of GDD (from transplanting to flowering, 89 days), respectively. Our results demonstrated that rice grain yield was more reduced by weed interference under elevated temperature conditions under which weed competitiveness increases, but the resultant growth of rice decreases more than under ambient conditions. Therefore, proper weed management needs to be performed to prevent rice yield loss from both weed interference and elevated temperatures.

**Table 4:** Parameter estimates for the combined rectangular hyperbolic model of rice grain yield as a result of single-weed interference caused by late watergrass and water chestnut at accumulated growing degree days (from transplanting to flowering, 89 days) in the pooled 3-year data.

	**Parameter estimates for the combined rectangular hyperbolic model^a^**						
**Weed species**	***y***_***oi***_			**β_***i***_**		**RMS**	**Pseudo-R^2^**
	***a***	***b***	***c***	***l***	***m***		
Late watergrass	−16.0 (10.00)	0.03 (0.0128)	−0.00001 (0.000004)	−0.021 (0.0152)	0.00002 (0.000010)	0.84	0.71
Water chestnut	−33.4 (8.97)	0.05 (0.0115)	−0.00002 (0.000004)	−0.031 (0.0096)	0.00003 (0.000007)	0.80	0.68

a *A combined rectangular hyperbolic model $\left(Y=\frac{a+bGDD+cGDD^{2}}{1+\left(l+mGDD\right)X}\right)$, where a, b, and c are unknown parameters for weed-free rice grain yield; l and m are unknown parameters for weed competitiveness; X is initial single weed density; and GDD is the accumulated growing degree days under elevated temperature. The numbers in parentheses are standard errors*.

## Discussion

### Effects of Elevated Temperature on Rice-Weed Competition

Our results clearly demonstrated that year, elevated temperature, and weed interference interactively affected rice during the entire growing season, but each effect on rice yield components was different. Weed interference more strongly reduced rice vegetative growth and its yield component (e.g., number of panicles) determined at earlier growth stages, while the interactive effect of year and elevated temperature more strongly reduced rice reproductive growth and its yield components (e.g., number of grains, % ripened grain, 1,000-grain weight) determined at later growth stages (Supplementary Table 1; Figure 1). Previous studies reported that the dry weight and number of panicles in rice are determined earlier than other yield components because early rice is the most susceptible to weed interference (Moon et al., 2010, 2014). Other yield components related to grains, such as the number of grains, % ripened grain, and 1,000-grain weight, are the most susceptible to elevated temperature during the late growth stage of rice, particularly from the heading to flowering stages (Jung et al., 2015). Our study also revealed that rice grain yield was reduced more significantly as a result of decreased spikelet fertility and 1,000-grain weight due to the elevated temperature, resulting in a greater than 30°C mean air temperature during the heading and flowering periods. Plotting the % ripened grain against the mean air temperature during its heading and flowering periods revealed a significant decrease in the % ripened grain from the point when the mean air temperature increased over 30°C, resulting in a logistic decrease with increasing mean air temperature (Supplementary Figure 2). Previous studies also reported that high air temperatures over 30°C due to elevated temperature affect spikelet fertility and grain filling, resulting in a reduced % ripened grain and 1,000-grain weight, respectively (Kim et al., 2011; Nguyen et al., 2014). Therefore, plotting the observed weed-free rice grain yield against GDD resulting from elevated temperature showed continuous grain yield responses to GDD, and this relationship can be described, using the quadratic model, as shown in Figure 4.

Weed competitiveness was enhanced by elevated temperature, but its effects on rice grain yield were mainly due to a decreased number of panicles (Supplementary Figure 3). Late watergrass was more competitive than water chestnut at ambient temperature, but the response of weed competitiveness to elevated temperature was similar (Figure 5). Late watergrass has greater competitiveness against rice than water chestnut, as it intercepts more photosynthetically active radiation due to its high canopy height (Bouhache and Bayer, 1993; Lindquist and Kropff, 1996). In contrast, water chestnut has shown variable competitiveness under different environmental conditions, depending on the soil type and its relative seedling emergence time (Kobayashi and Ueki, 1983; Chun and Shin, 1994). With respect to elevated temperature, weed competitiveness was not significantly different between late watergrass and water chestnut, showing the same linear increase in their competitiveness against rice with GDD (Figure 5, Supplementary Table 3). This finding thus suggests that the increase in weed competitiveness with GDD can be described, using the linear model, and can be incorporated into the model describing rice-weed competition on rice grain yield.

### Model Application for Future Weed Management

The combined model developed here can provide predictive information for rice grain yield caused by both elevated temperature and weed interference under future climate conditions. Our results indicated that the weed-free rice grain yield would increase by up to 5.8 t ha^−1^ at a GDD of 1,500 (°C days) and, thereafter, decrease with increasing GDD (Figure 6). The decrease in the weed-free rice grain yield was mainly attributed to high temperature-induced spikelet sterility during its heading and flowering periods (Table 1, Supplementary Figure 2). Similar results were previously reported for spikelet sterility of rice (Nguyen et al., 2014; Jung et al., 2015). The quadratic function of our combined model accurately described the impact of elevated temperature on the weed-free rice grain yield in the pooled 3-year data. The quadratic model represents the temperature effect on the weed-free yield of crops, including rice (Sheehy et al., 2006a,b). Our combined rectangular hyperbolic model based on GDD may propose a potential period for safe rice cultivation in consideration of weed interference under future elevated temperature conditions. However, the combined rectangular hyperbolic model needs to be further improved to describe the complex effects of elevated temperature on the spikelet sterility of rice during its heading and flowering periods. With respect to weed competitiveness, our results indicated that late watergrass and water chestnut can increase their competitiveness with an increase in the daily mean air temperature under future elevated temperature conditions. A linear function of our combined rectangular hyperbolic model provided an accurate description of the weed competitiveness response to elevated temperature. A study on weed competitiveness reported that the linear model accurately explained the response of weed competitiveness to other environmental factors, such as nitrogen fertilizer, in wheat (Kim et al., 2006). Weed competitiveness in our study increased less with increasing elevated temperature than the competitiveness affected by nitrogen fertilizer (e.g., Kim et al., 2006; Song et al., 2021). Although the elevated temperature had fewer effects on both crop growth and weed biomass than nitrogen fertilizer, the temperature effects on the interference relationships were quite considerable. Water chestnut was a less competitive weed species than late watergrass under ambient conditions but increased its competitiveness over rice to a value similar to the competitiveness for late watergrass under elevated temperature. Water chestnut can outcompete rice through root inhibition due to various factors, such as the dispersion of tuber propagation, earlier seedling emergence, and faster growth development under elevated temperature, although it is not a C4 weed species (Kim et al., 2010). In contrast, the same *Echinochloa* species as late watergrass showed a slight increase in aboveground biomass compared with the biomass of rice under elevated temperature (Alberto et al., 1996). These results indicated that the interference relationships under elevated temperature are dependent on the interactions among crop species, weed species, and air temperature during the growing seasons. Further validation of the combined rectangular hyperbolic model might be required to describe the complex effects of elevated temperature on rice-weed competition, using a more independent data set under various locations and years. Although the combined rectangular hyperbolic model and estimated parameters in this study need to be further validated, our combined model aids decision-making for timely weed control at the early rice growth stage under future elevated temperature conditions.

## Conclusion

The combined rectangular hyperbolic model developed by incorporating the quadratic model for weed-free rice grain yield and the linear model for weed competitiveness under elevated temperature conditions accurately describes both the effects of elevated temperature and weed interference on rice grain yield over 3 years. Our combined model can support decision-making for late watergrass and water chestnut management in rice production under future elevated temperature conditions. Our results demonstrate that rice grain yield is significantly reduced by weed interference under elevated temperature. Therefore, proper weed management based on a prediction model is required to prevent rice yield loss from both weed interference and elevated temperature. For example, rice should not only be cultivated to avoid spikelet sterility under future elevated temperature conditions but should also be kept weed free by timely weed control at its early growth stages. Further studies should be conducted to validate the parameters of the combined rectangular hyperbolic model for spikelet sterility of rice during its heading and flowering periods and enhanced weed competitiveness during the vegetative stage of rice as a result of elevated temperature. Although further validation in a broad range of years and locations is required for the combined rectangular hyperbolic model and estimated parameters, our study can provide empirical information for describing both the effects of elevated temperature and weed interference on crop yield and give decision support for weed management under a future climate.

## Data Availability Statement

The raw data supporting the conclusions of this article will be made available by the authors, without undue reservation.

## Nomenclature

Late watergrass, *Echinochloa oryzicola* Vasing.; water chestnut, *Eleocharis kuroguwai* Ohwi; ambient temperature, A; and accumulated growing degree days, GDD

## Author Contributions

J-SS, J-HI, and Y-HP were involved in conducting the experiments, gathering data, performing statistical analyses, interpretation of data, developing the figures and tables, and drafting the manuscript. D-SK was involved in designing the experiments and supervising the research as well as the overall revision of the manuscript. S-HL, M-JY, J-WK, and B-WL were involved in the revision of the manuscript. All the authors approved the final version of the manuscript.

## Conflict of Interest

The authors declare that the research was conducted in the absence of any commercial or financial relationships that could be construed as a potential conflict of interest.
