# Supplementary material for: Modeling the Effects of Elevated Temperature and Weed Interference on Rice Grain Yield
Source: Front Plant Sci. 2021 Jul 16;12:663779. doi: 10.3389/fpls.2021.663779 (PMC8330814; doi:10.3389/fpls.2021.663779)
Supplement: Supplementary file 1 [file Data_Sheet_1.docx]

**Supplementary materials**

**FIGURE S1** Temporal changes in daily mean temperature at four sunlit temperature-controlled phytotrons adjusted to ambient (A), A+1.5 °C, A+3.0 °C, and A+5.0 °C during rice growing period in 2014, 2015, and 2016.

**FIGURE S2** The percentage of ripened grains as a function of mean air temperature during the heading and flowering periods (11–28 August) in 2014, 2015, and 2016. The line is fitted values calculated using the logistic model.

**FIGURE S3** Weed competitiveness to number of panicles as a function of weed density of late watergrass (A) and water chestnut (B) with increasing accumulated growing degree days (from transplanting to flowering, 89 days) in 2014, 2015, and 2016. The lines are fitted values calculated using the linear model.
