# Supplementary material for: Modeling the Effects of Elevated Temperature and Weed Interference on Rice Grain Yield
Source: Front Plant Sci. 2021 Jul 16;12:663779. doi: 10.3389/fpls.2021.663779 (PMC8330814; doi:10.3389/fpls.2021.663779)
Supplement: Supplementary file 2 [file Data_Sheet_2.docx]

**Supplementary materials**

**TABLE S1** Summary of analysis of variance (ANOVA) for weed biomass, rice yield components, and grain yield as a result of single-weed interference of late watergrass and water chestnut at four sunlit temperature-controlled phytotrons adjusted to ambient, +1.5, +3.0, and +5.0 °C in 2014, 2015, and 2016. The data were analyzed with year (Y), elevated temperature (T), and weed density (W) as main factors and the replicate as a random factor. Significance is indicated as follows: ns, not significant; *, *P*<0.05; **, *P* <0.01; ***, *P* <0.001.

| Weed species | Source of variation | Degree of freedom | Weed Biomass  (g) | Rice yield components and grain yield | | | | | |
| --- | --- | --- | --- | --- | --- | --- | --- | --- | --- |
|  |  |  |  | Dry weight  (g) | No of panicles | No of grains | % ripened grain | 1000-grain weight (g) | Grain yield  (t ha^-1^) |
| Late watergrass | Y | 2 | *** | *** | *** | ns | ** | ns | *** |
|  | T | 3 | ns | ** | *** | *** | *** | *** | *** |
|  | W | 5 | *** | *** | *** | *** | ns | *** | *** |
|  | Y×T | 6 | ns | ** | *** | *** | *** | *** | *** |
|  | Y×W | 10 | ns | *** | ns | * | ns | ns | ** |
|  | T×W | 15 | ns | ns | ns | ** | ns | *** | *** |
|  | Y×T×W | 30 | ns | ns | ns | * | ns | ns | *** |
| Water chestnut | Y | 2 | *** | *** | ns | * | *** | *** | *** |
|  | T | 3 | ** | ns | ns | *** | *** | *** | *** |
|  | W | 5 | *** | *** | *** | * | ns | ns | *** |
|  | Y×T | 6 | ns | *** | ns | *** | *** | ** | *** |
|  | Y×W | 10 | *** | *** | ns | ns | * | ns | * |
|  | T×W | 15 | ns | ns | * | ns | ns | ns | * |
|  | Y×T×W | 30 | ns | ns | ns | ns | ns | ns | * |

**TABLE S2** Parameter estimates for the quadratic model for the regression of weed-free rice grain yield (*Y_o_*) affected by accumulated growing degree days (from transplanting to flowering, 89 days) in the pooled 3-year data obtained in Table 2.

| Weed species | Parameter estimates for weed-free rice grain yield ^a^ | | | RMS | Pseudo-R^2^ |
| --- | --- | --- | --- | --- | --- |
|  | *a* | *b* | *c* |  |  |
| Late watergrass | -32.4 (33.72) | 0.055 (0.0425) | -0.00002 (0.000013) | 1.43 | 0.53 |
| Water chestnut | -46.6 (33.78) | 0.073 (0.0426) | -0.00003 (0.000013) | 1.43 | 0.52 |

^a^ A quadratic model for weed-free rice grain yield ($Y_{0}=a+bGDD+c{GDD}^{2})$, where *a*, *b*, and *c* are unknown parameters and *GDD* is the accumulated growing degree days. The numbers in parentheses are standard errors.

**TABLE S3** Parameter estimates for the linear model for the regression of weed competitiveness (*β*) affected by accumulated growing degree days (from transplanting to flowering, 89 days) in the pooled 3-year data obtained in Table 2.

| Weed species | Parameter estimates for weed competitiveness ^a^ | | RMS | Pseudo-R^2^ |
| --- | --- | --- | --- | --- |
|  | *l* | *m* |  |  |
| Late watergrass | -0.03 (0.021) | 0.00003 (0.000013) | 0.006 | 0.30 |
| Water chestnut | -0.05 (0.015) | 0.00003 (0.000010) | 0.005 | 0.59 |

^a^ A linear model for weed competitiveness ($\beta=l+mGDD)$, where *l* and *m* are unknown parameters and *GDD* is the accumulated growing degree days. The numbers in parentheses are standard errors.
